# MicroRNA-23b Functions as a Tumor Suppressor by Regulating Zeb1 in Bladder Cancer

**DOI:** 10.1371/journal.pone.0067686

**Published:** 2013-07-02

**Authors:** Shahana Majid, Altaf A. Dar, Sharanjot Saini, Guoren Deng, Inik Chang, Kirsten Greene, Yuichiro Tanaka, Rajvir Dahiya, Soichiro Yamamura

**Affiliations:** 1 Department of Urology, VA Medical Center and UCSF, San Francisco, California, United States of America; 2 California Pacific Medical Center Research Institute, San Francisco, California, United States of America; University of Central Florida, United States of America

## Abstract

MicroRNAs (miRNAs) are small, non-coding RNAs that regulate gene expression by targeted repression of transcription and translation. In this study we show that miRNA-23b (miR-23b) acts as a tumor suppressor in bladder cancer. Quantitative real-time PCR analysis showed that miR-23b is significantly down-regulated in bladder cancer cell lines and tumor tissues compared to non-malignant cells and normal tissue samples. We also demonstrate that miR-23b expression has a potential to be diagnostic and prognostic biomarker in bladder cancer. High miR-23b expression is positively correlated with higher overall survival of bladder cancer patients as revealed by Kaplan-Meier analysis. ROC analysis showed that miR-23b expression can distinguish between normal and bladder cancer tissues. Further we elucidated the biological significance of miR-23b in bladder cancer. Over-expression of miR-23b in bladder cancer cells inhibited cell proliferation and impaired colony formation. Fluorescence activated cell sorting (FACS) analysis revealed that re-expression of miR-23b in bladder cancer cells induced G0/G1 cell cycle arrest and apoptosis while inhibiting cell migration and invasion. Luciferase reporter assays demonstrated that Zeb1, a crucial regulator of epithelial-to-mesenchymal transition (EMT), is a direct target of miR-23b in bladder cancer. These results show that loss of miR-23b confers a proliferative advantage and promotes bladder cancer cell migration and invasion. Furthermore, re-expression of miR-23b may be a beneficial therapeutic strategy for the treatment of human bladder cancer.

## Introduction

Bladder cancer is the fourth most common malignancy in the United States and one of the costliest to clinically manage [1]. More than 90% of urinary bladder tumors are comprised of transitional cell carcinoma (TCC) that arises from transitional epithelium [2]. Urinary bladder tumors are classified into two distinct categories: non-muscle and muscle invasive bladder cancer [3], [4]. Most tumors (75–80%) present as low-grade papillary non-invasive tumors that rarely progress to become lethal but almost always recur. This type of cancer is called “superficial” bladder cancer and requires expensive long-term management. The rest are high-grade muscle invasive tumors (∼15%) that can rapidly progress to become metastatic and lead to death [4]. Etiological factors involved in bladder carcinogenesis remain unidentified, and effective molecular markers for the disease are limited.

MicroRNAs (miRNAs) are small, non-coding RNAs that regulate gene expression by targeted repression of transcription and translation. Several studies have done a global analysis of miRNA expression in human cell lines and found tissue and disease-specific expression patterns [5], [6]. There is also increasing evidence that miRNA expression profiles may be indicative of disease risk and burden. Thus, miRNAs are being assessed as potential biomarkers to aid in the diagnosis and prognosis of different types of cancers [7], [8]. Several human miRNAs have been shown to be dysregulated in bladder cancer, including miR-1280, miR-203, miR-125b and miR-133a [9], [10], [11], [12] and contribute to the development and progression of the disease. Here we report that miR-23b is significantly down-regulated in bladder cancer tissues and cell lines and that high expression level of miR-23b positively correlate with higher overall survival of patients after surgery. In addition, we examined the functional significance of miR-23b and identified Zeb1 as a direct target of miR-23b in bladder cancer. For the first time this study shows that miR-23b is a potential biomarker and tumor suppressor in bladder cancer directly targeting oncogene Zeb1.

## Materials and Methods

### Cell Lines and Cell Culture

SV-HUC-1, T24 and J82 cells were purchased from the American Type Culture Collection (ATCC) and grown according to ATCC protocols. These human-derived cell lines were authenticated by DNA short-tandem repeat analysis by ATCC. The experiments with cell lines were performed within 6 months of their procurement/resuscitation. SV-HUC-1 cells were cultured in F-12K Medium (ATCC) with 10% FBS. T24 cells were cultured in McCoy’s 5A medium supplemented with 10% FBS and J82 cells were cultured in Minimum Essential Media (MEM) supplemented with 10% FBS. Cells were maintained in an incubator with a humidified atmosphere of 95% air and 5% CO_2_ at 37°C.

### Plasmids, Precursors and Transfection

TaqMan probes and precursors for hsa-miR-23b and negative control pre-miR were purchased from Applied Biosystems (Foster City, CA). pmir-GLO Dual-Luciferase miRNA Target Expression Vector was purchased from Promega. microRNA-23b, control-microRNA and siRNAs were used at 50 nM concentration and Lipofectamine 2000 (Invitrogen) was used for all transfections.

### RNA Extraction

miRNA and total RNA were extracted from cell lines using a miRNeasy Mini Kit and an RNeasy Mini Kit (Qiagen). miRNAs from clinical samples were extracted using laser capture microdissection techniques with a miRNeasy FFPE kit (Qiagen).

### Human Clinical Samples

Clinical samples were obtained from the San Francisco Veterans Affairs (VA) Medical Center. Written informed consent was obtained from all patients and the study was approved by the UCSF Committee on Human Research (Approval number: H9058-35751-01).

### Quantitative Real-time PCR

Mature miRNAs were assayed using TaqMan MicroRNA Assays in accordance with the manufacturer’s instructions (Applied Biosystems). All RT reactions, including no-template controls and RT minus controls, were run in a 7500 Fast Real Time PCR System (Applied Biosystems). RNA concentrations were determined with a NanoDrop (Thermo Scientific, Rockford, IL). Samples were normalized to RNU48 (Applied Biosystems). Gene expression levels were quantified using the 7500 Fast Real Time Sequence detection system Software (Applied Biosystems). Comparative real-time PCR was performed in triplicate, including no-template controls. Relative expression was calculated using the comparative Ct.

### Cell Viability and Clonability Assays

Cell viability was determined at 24, 48 and 72 h by using the CellTiter 96 AQueous One Solution Cell Proliferation Assay kit (Promega, Madison, WI) according to the manufacturer’s protocol. Absorbance was measured at 490 nm using SpectraMAX 190 (Molecular Devices). Data are presented as the mean value for triplicate experiments compared to the negative control. For colony formation assay, cells were seeded at low density (1000 cells/plate) and allowed to grow until visible colonies appeared. Then, cells were stained with Giemsa and colonies were counted.

### Migration and Invasion Assays

Cytoselect 24-well cell migration and invasion assay kits (Cell Biolabs, Inc) were used for migration and invasion assays according to the manufacturer’s protocol. Briefly, T24 and J82 cells transfected with Pre-miR miRNA precursor or negative control were harvested 72 hours after transfection and re-suspended in serum-free Opti-MEM. Cells (10×10^4^ per 300 µl media without serum) were added to the upper chamber, and the lower chamber was filled with 500 µl of media containing 10% FBS. Cells were incubated for 16 hours at 37°C in a 5% CO_2_ incubator. After 16 hours, non-migrated/non-invading cells were removed from upper side of trans-well membrane filter inserts using a cotton-tipped swab. Migrated/invaded cells on the lower side were stained and the absorbance was read at 560 nm according to the manufacturer’s protocol.

### Immunoblotting

Protein was isolated from confluent (70–80%) plates of cultured cells using the M-PER Mammalian Protein Extraction Reagent (Pierce Biotechnology, Rockfield, IL) following the manufacturer’s directions. Protein concentrations were determined by the Bradford method. Equal amounts of protein were resolved on 4–20% sodium dodecyl sulfate (SDS) polyacrylamide gels and transferred to a nitrocellulose membrane by voltage gradient transfer. The resulting blots were blocked with 5% non-fat dry milk and probed with specific antibodies. Blots were then incubated with appropriate peroxidase-conjugated secondary antibodies and visualized using enhanced chemiluminescence (Pierce Biotechnology, Rockford, IL).

### Depletion of Zeb1 Using Small Interfering RNA (siRNA)

T24 bladder cancer cells were plated 24 hours before transfection. At 40 to 50% confluence, cells were transfected using lipofectamine-2000 (Invitrogen, Carlsbad, CA) with siRNA duplexes specific for human Zeb1 (SR304746; Origene Technology, Rockville, MD) or control non-silencing (NS) siRNA for 72 hours. Initially, two different sets of siRNA duplexes were tested to evaluate the target specificity and knockdown efficiency. One siRNA duplex was used for further experiments at 50 nM concentration.

### Luciferase Reporter Assay

A pmirGLO Dual-Luciferase miRNA target expression vector was used for 3′-UTR luciferase assays (Promega, Madison, WI). The target oncogene of miRNA-23b was selected on the basis of online microRNA target database http://www.microrna.org/microrna/home.do. The primer sequences for the wild type 3′UTR were: Forward 5′ CGCGGCCGCTAGTATTATGTTTTTTAAAATGTGAGT 3′ and Reverse 5′ CTAGACTCACATTTTAAAAAACATAATACTAGCGGCCGCGAGCT 3′. For the mutant 3′UTR, the primer sequences were: Forward 5′ CGCGGCCGCTAGTATCGTGCGCACTAAGGCTCACTT 3′ and reverse 5′ CTAGAAGTGAGCCTTAGTGCGCACGATACTAGCGGCCGCGAGCT 3′. For lucifease assay, T24 and J82 cells were cotransfected with hsa-miR-23b and pmirGLO Dual-Luciferase miRNA target expression vectors with wild-type or mutant target sequence using Lipofectamine 2000. Firefly luciferase activities were measured using the Dual Luciferase Assay (Promega, Madison, WI) 18 hr after transfection and the results were normalized with Renilla luciferase. Each reporter plasmid was transfected at least three times (on different days) and each sample was assayed in triplicate.

### Statistical Analysis

Statistical analyses were performed with GraphPad Prism 5 and MedCalc version 10.3.2. All quantified data represents an average of at least triplicate samples or as indicated. Error bars represent standard deviation of the mean. All tests were performed two tailed and *p-*values <0.05 were considered statistically significant. Receiver operating curves (ROC) were calculated to determine the potential of miR-23b to discriminate between malignant and non-malignant samples. For survival analysis, Kaplan-Meier (log-rank test) analysis was performed.

## Results

### miR-23b Expression is Depleted in Bladder Tumors and Cancer Cell Lines

**Figure 1:**
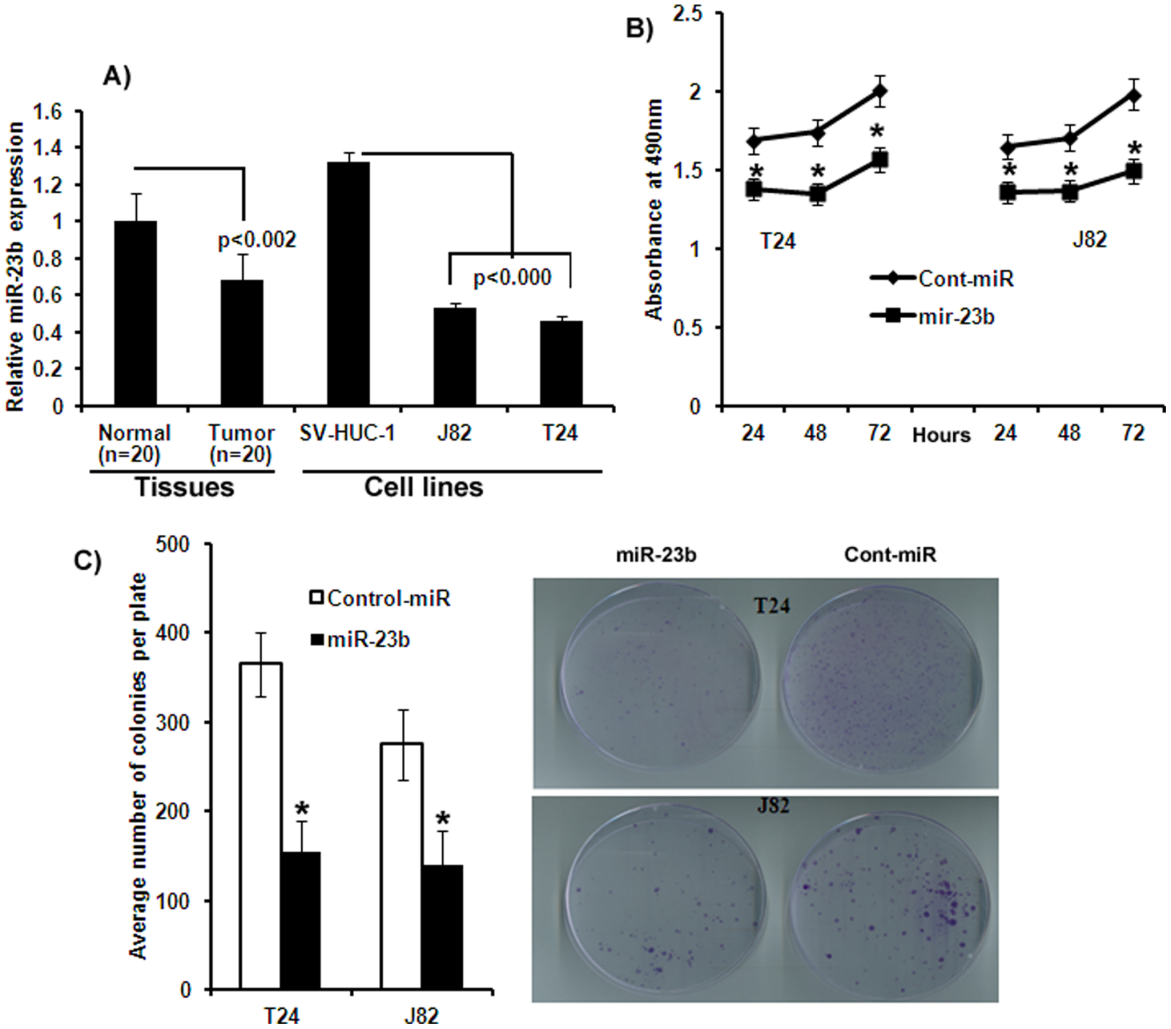
miR-23b expression profile and antiproliferative effect in bladder cancer. A) Quantitative RT-PCR analysis of miR-23b in cell lines and in matched laser-captured microdissected tissue samples. B) Proliferation of J82 and T24 cells after miR-23b transfection was significantly reduced compared to cont-miR. C) miR-23b over-expression significantly inhibits colony forming ability of bladder cancer cells.

Preliminary microRNA microarray data revealed that miR-23b was highly downregulated in bladder cancer cell lines compared to the non-malignant SV-HUC1 cell line. We validated the microarray data by miRNA-quantitative RT-PCR (miR qRT-PCR) analysis and results confirmed that miR-23b was downregulated in bladder cancer cell lines J82, T24 compared to non-malignant cell line SV-HUC1 (Figure 1A). To examine the biological relevance of miR-23b, its expression was analyzed in laser captured microdissected (LCM) human bladder tumor tissues and compared to normal matched control tissues. The expression of miR-23b was found to be significantly down-regulated in all the tumor samples compared to their matched normal samples (Figure 1A). Further the expression of miR-23b in normal tissues correlated with that of the non-malignant cell line and that of tumor correlated with the cancer cell lines (Figure 1A) indicating that these cancer cell lines represent a model system to analyze miR-23b function in bladder cancer. These results also suggest a putative tumor suppressor role for miR-23b in bladder cancer.

### MicroRNA-23b Regulates Bladder Cancer Cell Proliferation and Colony Formation

To determine the functional significance of miR-23b over expression in bladder cancer, we transfected bladder cancer cell lines J82 and T24 with miR-23b precursors. Ectopic expression of miR-23b significantly decreased cell proliferation as compared to cells expressing cont-miR (Figure 1B). miR-23b transfected cells had low colony formation ability as the number of foci in miR-23b expressing cells was decreased when compared with cont-miR transfected cells (Figure 1C). These results indicate anti-proliferative effect of miR-23b in bladder cancer.

### miR-23b Triggers Cell Cycle Arrest and Induces Apoptosis in Bladder Cancer Cells

**Figure 2:**
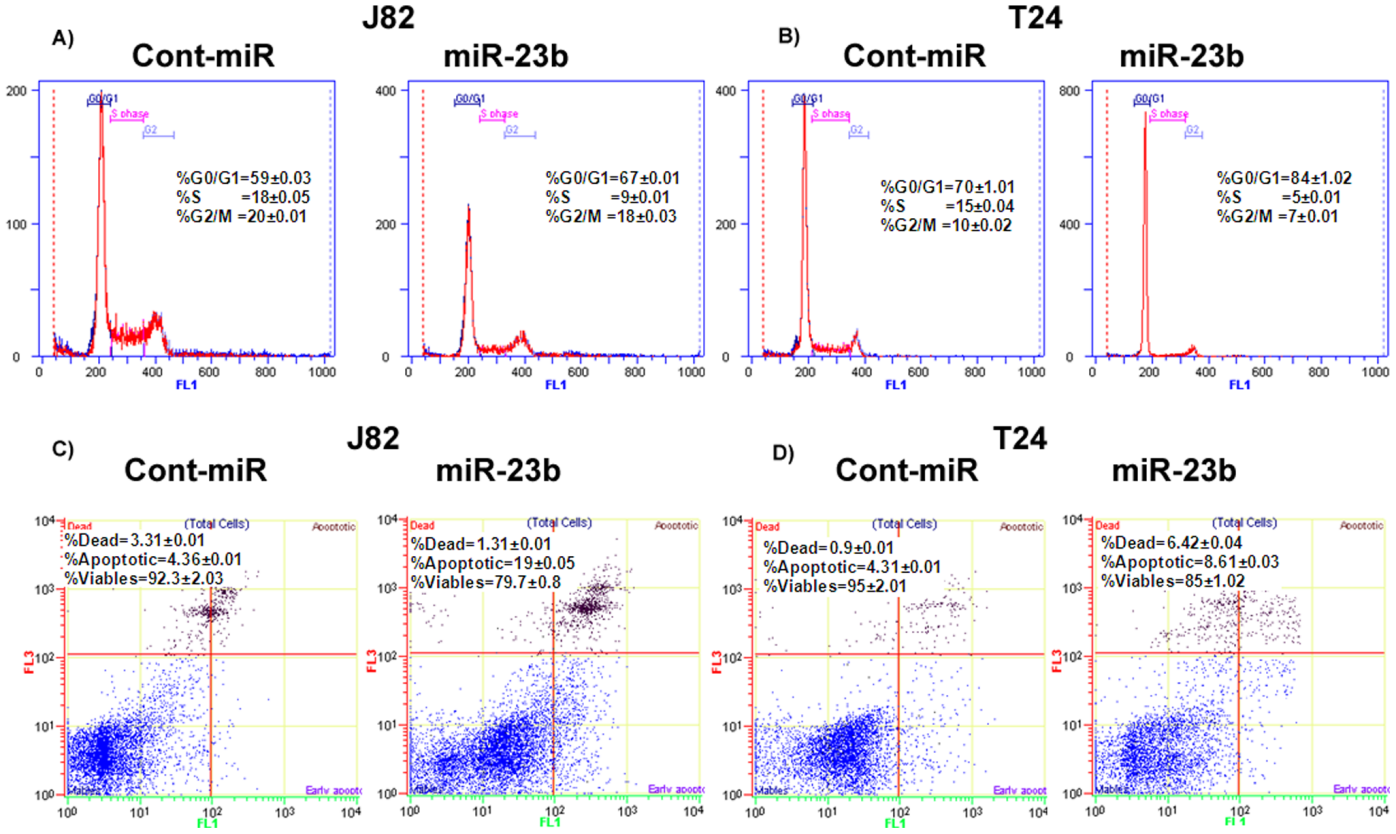
miR-23b induces cell cycle arrest and apoptosis in bladder cancer cells. A–B) Representative pictures of FACS analysis showing miR-23b over-expression induces G0/G1 cell cycle arrest in J82 and T24 cells with a corresponding decrease in S-phase cells. C–D) miR-23b over-expression induces apoptosis in J82 and T24 cells with a concomitant decrease in the viable number of cells. Data is shown from triplicate experiments ±SD.

FACS (fluorescence activated cell sorting) analysis revealed that re-expression of miR-23b led to a significant increase in the number of cells in the G0/G1 phase of the cell cycle (59% to 67%) while the S-phase population decreased from 18% to 9% in J82 cells (Figure 2A). Similar results were observed in T24 cells with an increase in the G0/G1 cell population (70% to 84%) and a decrease in S-phase population (15% to 5%) (Figure 2B). Thus suggesting that miR-23b triggers G0/G1 arrest in miR-23b transfected cells compared to cont-miR. FACS analysis for apoptosis was performed using Annexin-V-FITC-7-AAD dye. The percentage of total apoptotic cells (early apoptotic+apoptotic) was significantly increased (4% to 19%) in response to miR-23b over-expression compared to cont-miR with a corresponding 14% decrease in the viable cell population in J82 cells (Figure 2C). In T24 cells, an increase (4% to 9%) in apoptotic cells was observed with miR-23b over-expression compared to cont-miR (Figure 2D). These results indicate a tumor suppressor role for miR-23b in bladder cancer.

### miR-23b Suppresses Bladder Cancer Cell Migration and Invasion

**Figure 3:**
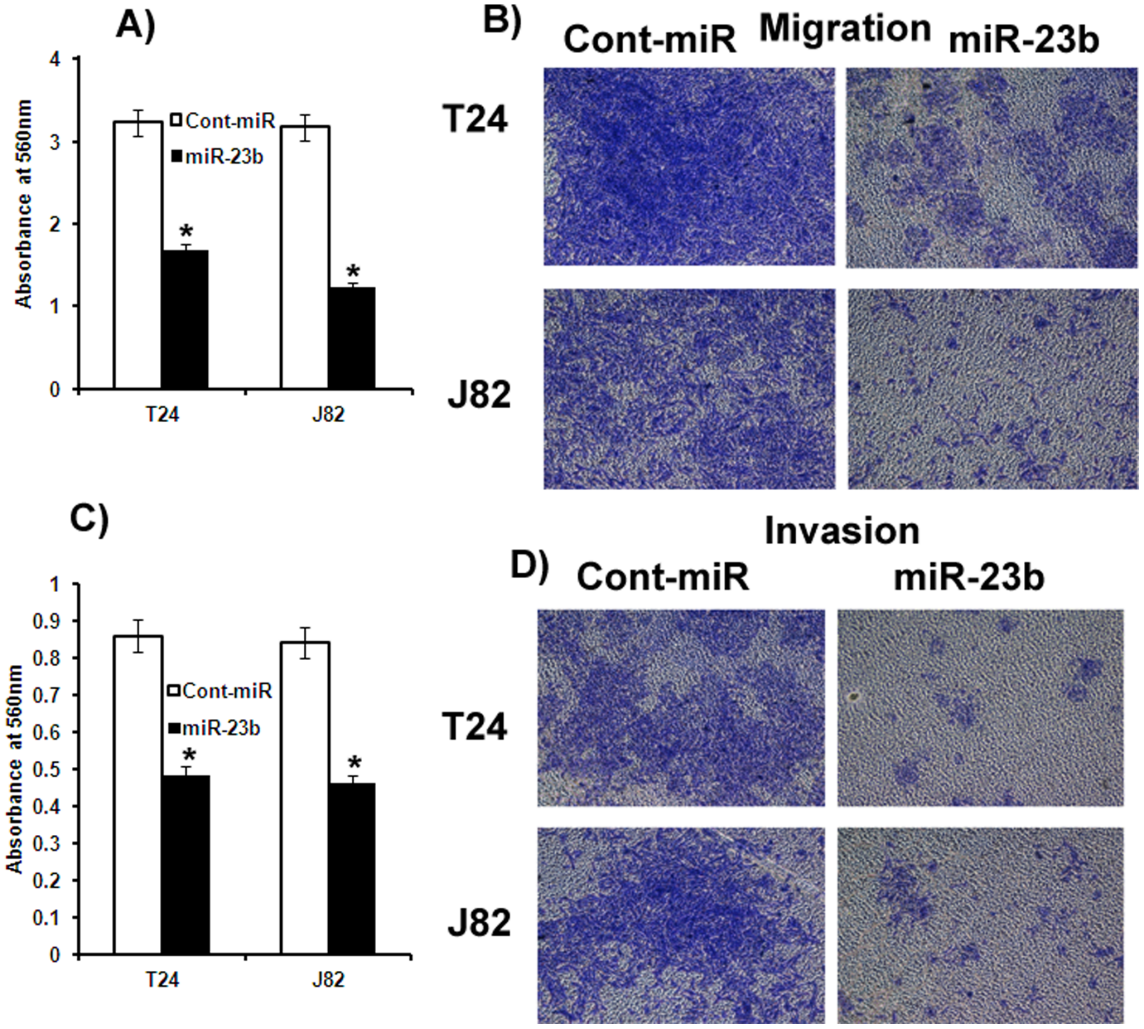
Ectopic expression of miR-23b inhibits bladder cancer cell migration and invasion. A) Migration assays of J82 and T24 cells transfected with miR-23b. B) Representative pictures of migration assay. C) Invasion assays show a significant decrease in the number of invading J82 and T24 cells transfected with miR-23b. D) Representative pictures of invasion assay.

Over-expression of miR-23b had anti-migratory and anti-invasive effects on bladder cancer cell lines. Less absorbance was observed at 560 nm with miR-23b transfected bladder cancer cells compared to cont-miR in the migration assay (Figure 3A) and miR-23b over-expression also significantly reduced the invasiveness of bladder cancer cells (Figure 3B).

### Oncogene Zeb1 is a Direct Target of miR-23b

**Figure 4:**
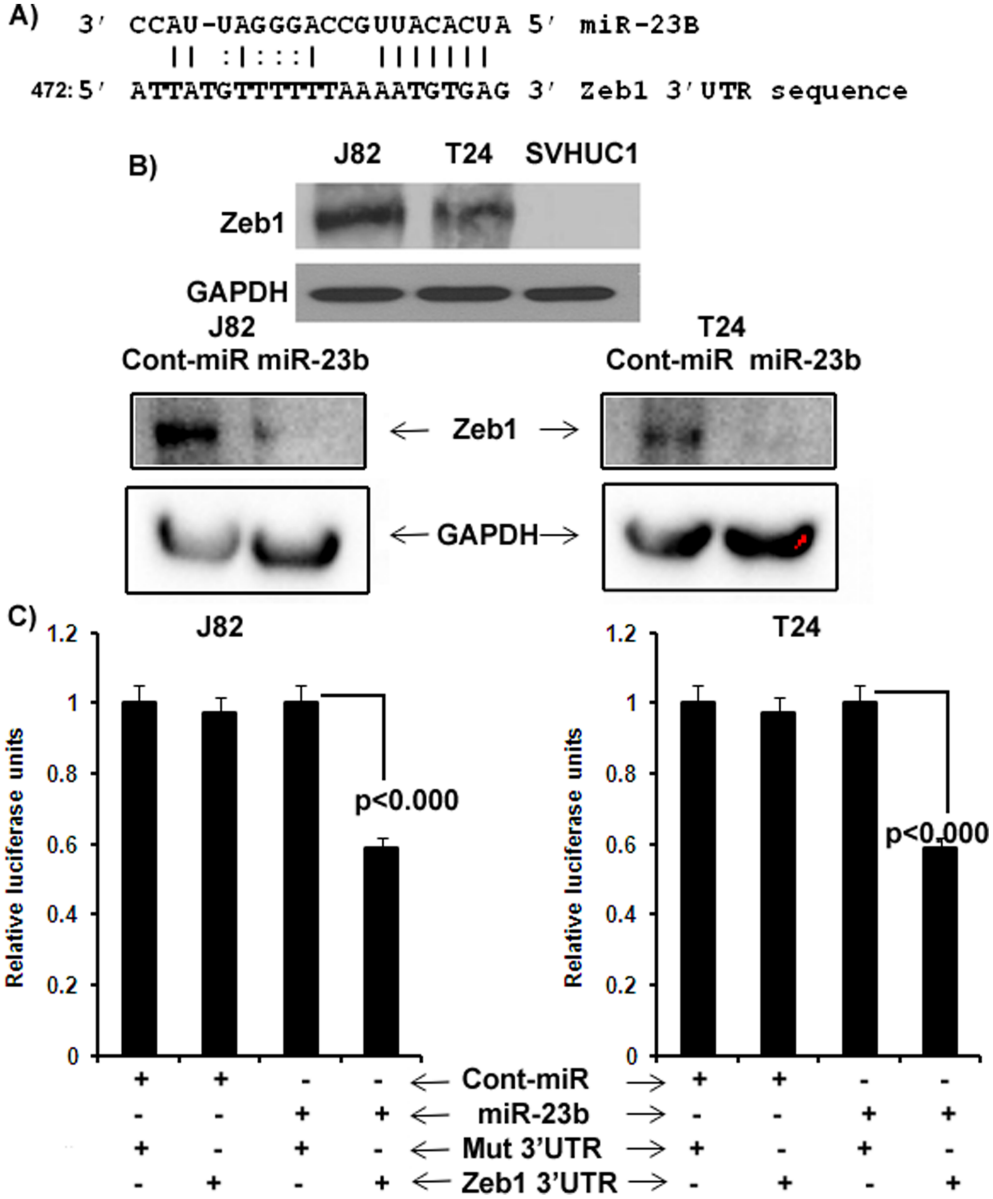
miR-23b directly targets EMT regulator Zeb1. A) Complimentary miR-23b binding sequences in the Zeb1 3′UTR. B) Western blot analysis shows that miR-23b represses translation of Zeb1 protein in J82 and T24 bladder cancer cells. C) Luciferase assays showing decreased reporter activity after co-transfection of either the wild type or mutant Zeb1-3′UTR with miR-23b in J82 and T24 cells. Mut- Mutated Zeb1 3′UTR sequence.

Zeb1 has been reported to be an important molecule that drives bladder cancer cell motility. Using an online microRNA target database we found oncogene Zeb1 to be a potential target of miR-23b with a complementary 3′UTR binding site for the seed sequence of miR-23b (Figure 4A). We performed Western analysis with miR-23b transfected cells and found that miR-23b attenuated expression of Zeb1 protein compared to cont-miR in both J82 and T24 bladder cancer cells (Figure 4B). To check whether a direct interaction is involved between miR-23b and its target oncogene Zeb1, we performed luciferase reporter assays. We found that co-transfection of miR-23b along with the wild type 3′UTR of Zeb1 caused a significant decrease in luciferase activity compared to controls (Figure 4C). These results suggest that miR-23b directly targets oncogene Zeb1.

### Depletion of Zeb1 by RNA Interference Mimics miR-23b Reconstitution in Bladder Cancer

**Figure 5:**
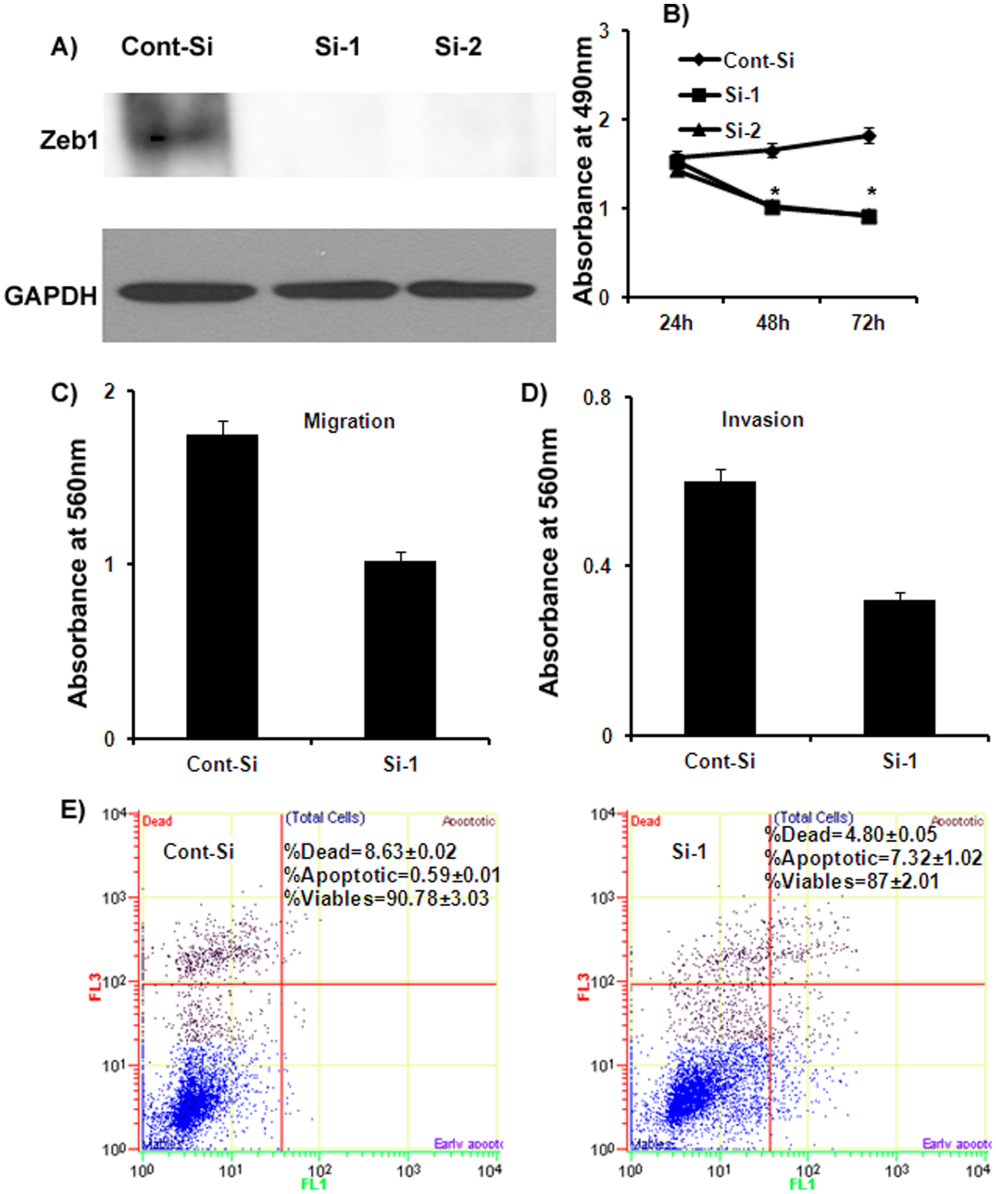
Depletion of Zeb1 by siRNA mimics miR-23b overexpression. A) Zeb1 protein levels were significantly attenuated with 50 nM siRNA duplexes (Si) compared to a non-silencing siRNA duplex (Con) in T24 cells. B) Effect on cell proliferation. C–D) Effect on migration and invasion of T24 bladder cancer cells. E) Apoptosis assay showing induction of apoptosis after Zeb1 knockdown by siRNA in T24 cells. *p<0.05.

Phenocopy experiments were also performed by siRNA inhibition of Zeb1 (Figure 5). We validated two sets of siRNA (Si-1 and Si-2) that resulted in significant knockdown of Zeb1 at protein level (Figure 5A) in T24 bladder cancer cells and used one siRNA duplex for further experiments at 50 nM concentration. Our results showed that siRNA inhibition of Zeb1 caused decreased cell viability (Figure 5B), migratory and invasive capability (Figure 5C–D) of T24 cancer cells. We also observed that siRNA inhibition of Zeb1 increased approximately 7% of the apoptotic fraction of cells in Zeb1 siRNA transfected cells compared to <1% in non-specific control (Figure 5E). These results suggest that siRNA depletion of Zeb1 mimics the effect of miR-23b over-expression in bladder cancer.

### Diagnostic and Prognostic Significance of miR-23b in Bladder Cancer

**Figure 6:**
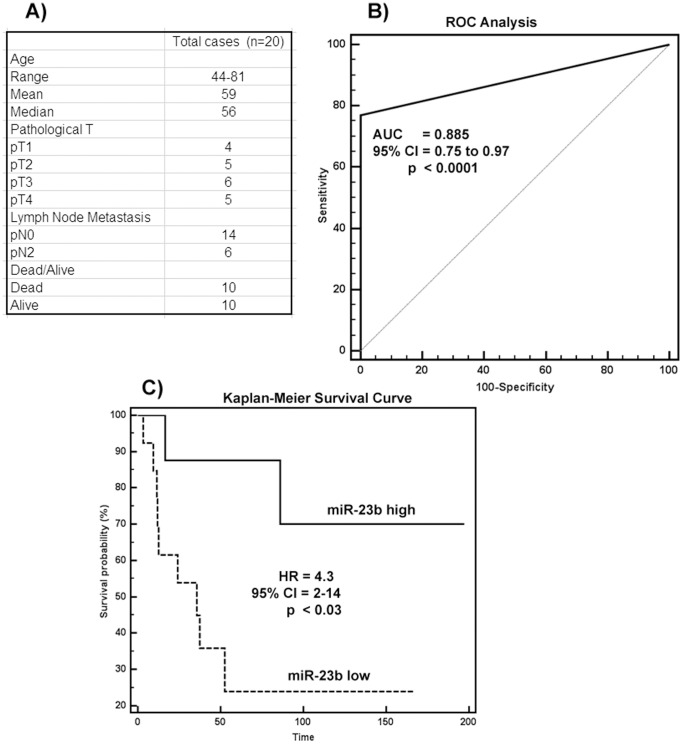
Diagnostic and prognostic significance of miR-23b in bladder cancer. A) Clinicopathological characteristics of patient cohort. B) ROC curve analysis showing ability of miR-23b expression to discriminate between malignant and non-malignant tissue samples. C) Kaplan-Meier analysis for overall survival based on miR-23b expression.

To determine whether miR-23b expression can discriminate between bladder tumors and normal tissues, and predict patient survival, we performed ROC analysis and Kaplan-Meier analysis. The clinical demographics of the patient cohort are summarized in Figure 6A. The area under the ROC curve (AUC) of 0.885 (P<0.0001; 95% CI = 0.75 to 0.97) (Figure 6B) suggested that miR-23b expression can discriminate between malignant and non-malignant tissues and potentially be used as a diagnostic marker for bladder cancer. To determine whether miR-23b has any prognostic significance, we divided patient tissues into low (expression T/N<1.2 fold) and high (expression T/N>1.2 fold) miR-23b groups and performed Kaplan-Meier survival analysis. Kaplan-Meier analysis showed that the high miR-23b group had significantly higher overall survival probability compared to the low miR-23b group (Logrank Test p<0.03, Hazard Ratio (HR) = 4.3, 95%CI = 2–14) (Figure 6C). These findings suggest that miR-23b is potentially a diagnostic and prognostic marker for bladder cancer though studies on additional samples are needed to strengthen these results.

## Discussion

MicroRNAs can have large-scale effects by regulating expression of a variety of genes during mammalian development and carcinogenesis. As a result, understanding the mechanisms and function of individual miRNAs has generated great interest. Despite the accumulating evidence regarding the role of various miRNAs in cancer, very limited information is available about the function of miRNAs in bladder cancer, and only few miRNA targets have been identified.

Here we report that miR-23b to be down-regulated in bladder cancer tissues compared to normal adjacent tissues and this was also observed in bladder cancer and non-malignant cell lines. Our data suggests a potential diagnostic/prognostic role for miR-23b in predicting overall survival and discriminating malignant from normal tissues and indicates that miR-23b is a tumor suppressor in bladder cancer.

To determine the biological relevance of miR-23b in bladder cancer, we performed functional assays. Ectopic expression of miR-23b resulted in significant inhibition of cell proliferation, colony formation, migration/invasion and induction of cell cycle arrest and apoptosis in bladder cancer cells. Expression of miR-23b in cancer is somewhat controversial because it has been found to be either up-regulated and oncogenic in kidney cancer where it caused translational repression of tumor suppressor PTEN gene [13] or down-regulated and a tumor suppressor in prostate cancer where it directly targets Src kinase and Akt oncogenes [14], while our study indicates it is a tumor suppressor in bladder cancer. Previous studies have shown that microRNAs are highly tissue specific and they can act as tumor suppressor or oncogenes [15], [16]. MicroRNAs possess several features that make them attractive candidates as new prognostic biomarkers and powerful tools for the early diagnosis of cancer [17]. In this study, we found that miR-23b was predictive of overall survival such that patients with higher miR-23b expression had longer overall survival compared to patients with low miR-23b expression. MicroRNA-23b expression was also able to distinguish malignant from normal tissues indicating the diagnostic significance of miR-23b in bladder cancer although additional studies with a larger cohort of tissue samples are required.

A significant obstacle to understanding miRNA function has been the relative paucity of experimentally validated targets. To determine the effectors of miR-23b, in-silico algorithms and functional analyses identified Zeb1 as its target. We demonstrated that miR-23b directly targets the 3′UTR of Zeb1, as its over-expression was associated with suppression of luciferase activity. In addition, a significant down-regulation in the level of Zeb1 protein was observed after miR-23b over-expression, indicating post-transcriptional regulation of Zeb1 via targeting its 3′UTR. Functional assays performed after Zeb1 depletion by siRNA transfection mimicked the results obtained with miR-23b overexpression. These results indicate that effects of miR-23b in bladder cancer are partly by directly targeting Zeb1, though other targets may also be involved since microRNAs can target thousands of genes. Zeb1 is one of the crucial regulators of epithelial-to-mesenchymal transition (EMT) [18] and has been shown to play a major role in invasion and metastasis of epithelial tumors [19]. The relevance of ZEB proteins to tumor progression has been studied in several human cancers. Expression of ZEB1 correlated with an aggressive phenotype in various histological types of endometrial carcinoma and was detected in the sarcomatous compartment of endometrial carcinosarcoma [20]. In colon cancer, ZEB1 was expressed at the invasive front of tumors, in association with the transient loss of basement membranes [21]. Reciprocal expression of ZEB1 and E-cadherin has also been observed in non-small cell lung carcinoma [22]. A direct correlation between ZEB1 immunoreactivity and Gleason grade has been reported in human prostate tumors [23] and in bladder cancer, ZEB1 has been reported to be over-expressed and responsible for enhanced motility [18]. In the present study, we found that over-expression of miR-23b resulted in suppression of oncogene Zeb1 in the bladder cancer cells suggesting that miR-23b can mediate EMT, thereby representing a possible mechanism through which it affects bladder cancer migration and invasion.

In conclusion, this study shows that miR-23b has diagnostic/prognostic significance and directly targets oncogenic Zeb1 in bladder cancer. miR-23b over-expression resulted in suppression of the bladder cancer cell proliferation and invasion, inducing apoptosis, and cell cycle arrest. Finally, this study indicates that miR-23b over-expression may be a therapeutically useful strategy for the treatment of bladder cancer.
